# 2-(2-Nitro­anilino)benzoic acid

**DOI:** 10.1107/S1600536811053529

**Published:** 2011-12-21

**Authors:** Xiao-Lin Zhu, Lu Shi, Peng Jiang, Tian-Hao Zhu, Hong-Jun Zhu

**Affiliations:** a Department of Applied Chemistry, College of Science, Nanjing University of Technology, Nanjing 210009, People’s Republic of China

## Abstract

In the title compound, C_13_H_10_N_2_O_4_, the nitro N atom deviates by 0.031 (2) Å from the plane of the benzene ring to which it is attached. The aromatic rings are oriented at a dihedral angle of 50.6 (1)°. An intra­molecular N—H⋯O hydrogen bond occurs. In the crystal, inversion dimers are formed by pairs of O—H⋯O inter­actions.

## Related literature

For the use of the title compound as an inter­mediate in the synthesis pharmacologically important compounds, see: Kelleher *et al.* (2007 ▶). For the synthesis, see: Rewcastle *et al.* (1987 ▶). For bond-length data, see: Allen *et al.* (1987 ▶).
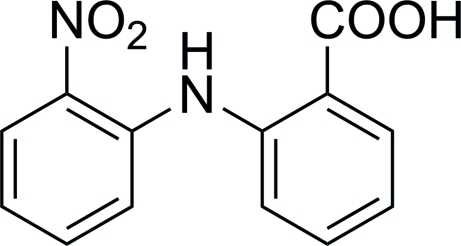


## Experimental

### 

#### Crystal data

C_13_H_10_N_2_O_4_
*M* *_r_* = 258.23
Monoclinic, 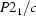

*a* = 7.1840 (14) Å
*b* = 21.546 (4) Å
*c* = 7.9070 (16) Å
β = 101.62 (3)°
*V* = 1198.8 (4) Å^3^
*Z* = 4
Mo *K*α radiation
μ = 0.11 mm^−1^
*T* = 293 K
0.30 × 0.20 × 0.10 mm

#### Data collection

Enraf–Nonius CAD-4 diffractometer
4704 measured reflections
2209 independent reflections
1437 reflections with *I* > 2σ(*I*)
*R*_int_ = 0.046
3 standard reflections every 200 reflections  intensity decay: 1%

#### Refinement

*R*[*F* ^2^ > 2σ(*F* ^2^)] = 0.057
*wR*(*F* ^2^) = 0.158
*S* = 1.01
2209 reflections
172 parameters
H-atom parameters constrained
Δρ_max_ = 0.19 e Å^−3^
Δρ_min_ = −0.32 e Å^−3^

### 

Data collection: *CAD-4 EXPRESS* (Enraf–Nonius, 1994 ▶); cell refinement: *SET4* (Enraf–Nonius, 1994 ▶); data reduction: *MolEN* (Harms & Wocadlo,1995 ▶); program(s) used to solve structure: *SHELXS97* (Sheldrick, 2008 ▶); program(s) used to refine structure: *SHELXL97* (Sheldrick, 2008 ▶); molecular graphics: *SHELXTL* (Sheldrick, 2008 ▶); software used to prepare material for publication: *SHELXTL*.

## Supplementary Material

Crystal structure: contains datablock(s) I, global. DOI: 10.1107/S1600536811053529/im2343sup1.cif

Structure factors: contains datablock(s) I. DOI: 10.1107/S1600536811053529/im2343Isup2.hkl

Supplementary material file. DOI: 10.1107/S1600536811053529/im2343Isup3.cml

Additional supplementary materials:  crystallographic information; 3D view; checkCIF report

## Figures and Tables

**Table 1:** Hydrogen-bond geometry (Å, °)

*D*—H⋯*A*	*D*—H	H⋯*A*	*D*⋯*A*	*D*—H⋯*A*
N1—H1*A*⋯O3	0.86	2.02	2.636 (3)	128
O1—H1*C*⋯O2^i^	0.82	1.82	2.636 (2)	176

Symmetry code: (i) 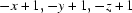.

**Fig. 1.**
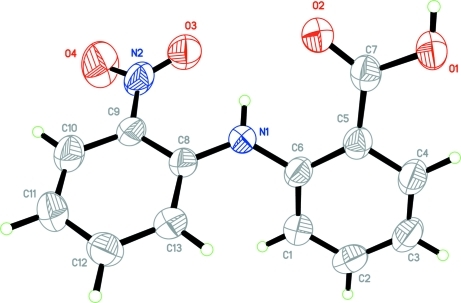
Molecular structure of (I), with displacement ellipsoids drawn at the 50% probability level.

**Fig. 2.**
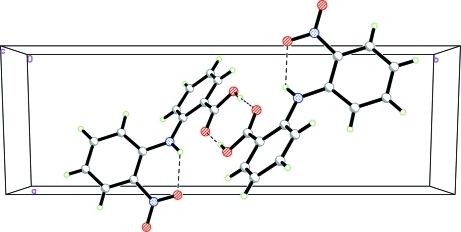
Packing diagram of (I). Hydrogen bonds are shown as dashed lines.

## supplementary crystallographic information

### Comment

The title compound, 2-(2-nitrophenylamino)benzoic acid is an important intermediate for the synthesis of 10,11-dihydro-5-acetyl-dibenzo[b,e][1,4]diazepin-11-one (Kelleher *et al.*, 2007). The crystal structure of the title compound, (I), is reported herein.

The molecular structure of (I) is shown in Fig. 1, and the intermolecular O—H···O hydrogen bond (Table 1) results in the formation of centrosymmetric carboxylic acid dimers. The bond lengths and angles are within normal ranges (Allen *et al.*, 1987).

In the molecule of the title compound, the rings are planar. The dihedral angle of the rings *Cg*1(C1—C6), *Cg*2(C8—C13) is: *Cg*1/*Cg*2 = 50.6 (1)°. The N atom is situated in the same plane as the phenyl ring to which it is attached.

In the crystal structure of the title compound, (I),  intra- and intermolecular O—H···O and N—H···O hydrogen bonds are observed. Centrosymmetrical dimers are formed by the O—H···O interaction.

### Experimental

The title compound, (I), was prepared by a literature method (Rewcastle *et al.*, 1987). Crystals suitable for X-ray analysis were obtained by dissolving (I) (0.20 g, 0.8 mmol) in acetone (25 ml) and evaporating the solvent slowly at room temperature for about 7 d.

### Refinement

H atoms were positioned geometrically and refined as riding groups, with O—H = 0.82 and C—H = 0.93 Å for aromatic H, and constrained to ride on their parent atoms, with *U*_iso_(H) = *xU*_eq_(C), where *x* = 1.2 for aromatic H, and *x* = 1.5 for other H.

### Crystal data

**Table:**

C_13_H_10_N_2_O_4_	*F*(000) = 536
*M**_r_* = 258.23	*D*_x_ = 1.431 Mg m^−^^3^
Monoclinic, *P*2_1_/*c*	Melting point: 490 K
Hall symbol: -P 2ybc	Mo *K*α radiation, λ = 0.71073 Å
*a* = 7.1840 (14) Å	Cell parameters from 25 reflections
*b* = 21.546 (4) Å	θ = 10–13°
*c* = 7.9070 (16) Å	µ = 0.11 mm^−^^1^
β = 101.62 (3)°	*T* = 293 K
*V* = 1198.8 (4) Å^3^	Block, yellow
*Z* = 4	0.30 × 0.20 × 0.10 mm

### Data collection

**Table:**

Enraf–Nonius CAD-4 diffractometer	*R*_int_ = 0.046
Radiation source: fine-focus sealed tube	θ_max_ = 25.4°, θ_min_ = 1.9°
graphite	*h* = 0→8
ω/2θ scans	*k* = −25→25
4704 measured reflections	*l* = −9→9
2209 independent reflections	3 standard reflections every 200 reflections
1437 reflections with *I* > 2σ(*I*)	intensity decay: 1%

### Refinement

**Table:**

Refinement on *F*^2^	Primary atom site location: structure-invariant direct methods
Least-squares matrix: full	Secondary atom site location: difference Fourier map
*R*[*F*^2^ > 2σ(*F*^2^)] = 0.057	Hydrogen site location: inferred from neighbouring sites
*wR*(*F*^2^) = 0.158	H-atom parameters constrained
*S* = 1.01	*w* = 1/[σ^2^(*F*_o_^2^) + (0.092*P*)^2^] where *P* = (*F*_o_^2^ + 2*F*_c_^2^)/3
2209 reflections	(Δ/σ)_max_ < 0.001
172 parameters	Δρ_max_ = 0.19 e Å^−^^3^
0 restraints	Δρ_min_ = −0.32 e Å^−^^3^

### Special details

**Table:**

Geometry. All e.s.d.'s (except the e.s.d. in the dihedral angle between two l.s. planes) are estimated using the full covariance matrix. The cell e.s.d.'s are taken into account individually in the estimation of e.s.d.'s in distances, angles and torsion angles; correlations between e.s.d.'s in cell parameters are only used when they are defined by crystal symmetry. An approximate (isotropic) treatment of cell e.s.d.'s is used for estimating e.s.d.'s involving l.s. planes.
Refinement. Refinement of *F*^2^ against ALL reflections. The weighted *R*-factor *wR* and goodness of fit *S* are based on *F*^2^, conventional *R*-factors *R* are based on *F*, with *F* set to zero for negative *F*^2^. The threshold expression of *F*^2^ > σ(*F*^2^) is used only for calculating *R*-factors(gt) *etc*. and is not relevant to the choice of reflections for refinement. *R*-factors based on *F*^2^ are statistically about twice as large as those based on *F*, and *R*- factors based on ALL data will be even larger.

### Fractional atomic coordinates and isotropic or equivalent isotropic displacement parameters (Å^2^)

**Table:**

	*x*	*y*	*z*	*U*_iso_*/*U*_eq_	
N1	0.3630 (3)	0.63169 (9)	0.8669 (2)	0.0512 (6)	
H1A	0.2975	0.6083	0.7889	0.061*	
O1	0.6904 (2)	0.49333 (8)	0.6877 (2)	0.0644 (6)	
H1C	0.6478	0.4799	0.5906	0.097*	
C1	0.5770 (4)	0.62113 (12)	1.1447 (3)	0.0525 (6)	
H1B	0.5117	0.6527	1.1882	0.063*	
O2	0.4326 (2)	0.55260 (8)	0.6238 (2)	0.0565 (5)	
N2	−0.0301 (3)	0.66483 (11)	0.7232 (3)	0.0617 (6)	
C2	0.7255 (4)	0.59204 (13)	1.2522 (3)	0.0584 (7)	
H2A	0.7594	0.6040	1.3673	0.070*	
O3	0.0167 (3)	0.61293 (10)	0.6871 (3)	0.0755 (6)	
C3	0.8248 (4)	0.54511 (14)	1.1905 (3)	0.0604 (7)	
H3A	0.9260	0.5257	1.2629	0.073*	
C4	0.7721 (3)	0.52775 (12)	1.0218 (3)	0.0550 (7)	
H4A	0.8376	0.4957	0.9809	0.066*	
O4	−0.1970 (3)	0.68049 (12)	0.6926 (4)	0.1088 (9)	
C5	0.6226 (3)	0.55667 (10)	0.9083 (3)	0.0437 (6)	
C6	0.5225 (3)	0.60428 (11)	0.9718 (3)	0.0447 (6)	
C7	0.5730 (3)	0.53451 (11)	0.7287 (3)	0.0469 (6)	
C8	0.2996 (3)	0.69134 (11)	0.8744 (3)	0.0428 (6)	
C9	0.1125 (3)	0.70950 (11)	0.8017 (3)	0.0461 (6)	
C10	0.0547 (4)	0.77113 (13)	0.8017 (3)	0.0599 (7)	
H10A	−0.0698	0.7817	0.7519	0.072*	
C11	0.1794 (4)	0.81632 (13)	0.8742 (4)	0.0618 (7)	
H11A	0.1414	0.8576	0.8723	0.074*	
C12	0.3632 (4)	0.79945 (11)	0.9505 (3)	0.0537 (7)	
H12A	0.4478	0.8296	1.0035	0.064*	
C13	0.4222 (3)	0.73919 (11)	0.9492 (3)	0.0492 (6)	
H13A	0.5473	0.7295	0.9993	0.059*	

### Atomic displacement parameters (Å^2^)

**Table:**

	*U*^11^	*U*^22^	*U*^33^	*U*^12^	*U*^13^	*U*^23^
N1	0.0457 (12)	0.0446 (12)	0.0561 (13)	0.0086 (10)	−0.0068 (9)	−0.0118 (9)
O1	0.0490 (11)	0.0629 (12)	0.0772 (13)	0.0150 (9)	0.0031 (9)	−0.0213 (9)
C1	0.0485 (14)	0.0528 (15)	0.0545 (15)	0.0053 (12)	0.0068 (11)	−0.0022 (12)
O2	0.0558 (11)	0.0512 (11)	0.0580 (10)	0.0111 (9)	0.0009 (8)	−0.0077 (8)
N2	0.0439 (13)	0.0625 (16)	0.0708 (15)	0.0036 (11)	−0.0069 (11)	0.0035 (12)
C2	0.0530 (15)	0.0641 (17)	0.0522 (15)	−0.0082 (14)	−0.0036 (12)	0.0016 (13)
O3	0.0606 (13)	0.0603 (13)	0.0932 (15)	−0.0053 (10)	−0.0141 (10)	−0.0106 (11)
C3	0.0407 (14)	0.0642 (18)	0.0697 (18)	0.0035 (12)	−0.0049 (13)	0.0077 (14)
C4	0.0385 (13)	0.0537 (16)	0.0705 (18)	0.0056 (12)	0.0051 (12)	0.0014 (12)
O4	0.0413 (12)	0.099 (2)	0.171 (3)	0.0063 (12)	−0.0146 (14)	−0.0119 (16)
C5	0.0347 (12)	0.0401 (13)	0.0557 (14)	−0.0018 (10)	0.0076 (10)	0.0007 (10)
C6	0.0358 (12)	0.0447 (14)	0.0507 (14)	0.0008 (10)	0.0016 (10)	0.0023 (10)
C7	0.0405 (13)	0.0360 (13)	0.0637 (16)	−0.0013 (11)	0.0095 (12)	−0.0001 (11)
C8	0.0408 (13)	0.0457 (14)	0.0407 (12)	0.0064 (10)	0.0053 (10)	−0.0035 (10)
C9	0.0399 (13)	0.0509 (15)	0.0445 (13)	0.0042 (11)	0.0013 (10)	−0.0017 (11)
C10	0.0501 (15)	0.0627 (18)	0.0640 (17)	0.0177 (14)	0.0043 (13)	0.0021 (13)
C11	0.0689 (19)	0.0462 (16)	0.0692 (18)	0.0145 (14)	0.0113 (14)	−0.0005 (13)
C12	0.0631 (16)	0.0470 (15)	0.0495 (14)	−0.0038 (13)	0.0081 (12)	−0.0047 (11)
C13	0.0434 (14)	0.0513 (16)	0.0494 (14)	0.0011 (11)	0.0013 (11)	−0.0036 (11)

### Geometric parameters (Å, °)

**Table:**

N1—C8	1.369 (3)	C3—H3A	0.9300
N1—C6	1.402 (3)	C4—C5	1.399 (3)
N1—H1A	0.8600	C4—H4A	0.9300
O1—C7	1.309 (3)	C5—C6	1.402 (3)
O1—H1C	0.8200	C5—C7	1.472 (3)
C1—C2	1.374 (3)	C8—C13	1.407 (3)
C1—C6	1.392 (3)	C8—C9	1.407 (3)
C1—H1B	0.9300	C9—C10	1.391 (3)
O2—C7	1.233 (3)	C10—C11	1.368 (4)
N2—O3	1.218 (3)	C10—H10A	0.9300
N2—O4	1.222 (3)	C11—C12	1.385 (4)
N2—C9	1.451 (3)	C11—H11A	0.9300
C2—C3	1.382 (4)	C12—C13	1.366 (3)
C2—H2A	0.9300	C12—H12A	0.9300
C3—C4	1.363 (3)	C13—H13A	0.9300
			
C8—N1—C6	127.5 (2)	C1—C6—C5	118.6 (2)
C8—N1—H1A	116.2	N1—C6—C5	120.9 (2)
C6—N1—H1A	116.2	O2—C7—O1	121.9 (2)
C7—O1—H1C	109.5	O2—C7—C5	123.5 (2)
C2—C1—C6	121.2 (2)	O1—C7—C5	114.6 (2)
C2—C1—H1B	119.4	N1—C8—C13	121.4 (2)
C6—C1—H1B	119.4	N1—C8—C9	122.9 (2)
O3—N2—O4	120.9 (2)	C13—C8—C9	115.7 (2)
O3—N2—C9	120.3 (2)	C10—C9—C8	121.7 (2)
O4—N2—C9	118.8 (2)	C10—C9—N2	116.6 (2)
C1—C2—C3	120.5 (2)	C8—C9—N2	121.6 (2)
C1—C2—H2A	119.8	C11—C10—C9	120.6 (2)
C3—C2—H2A	119.8	C11—C10—H10A	119.7
C4—C3—C2	119.0 (2)	C9—C10—H10A	119.7
C4—C3—H3A	120.5	C10—C11—C12	118.8 (2)
C2—C3—H3A	120.5	C10—C11—H11A	120.6
C3—C4—C5	122.1 (2)	C12—C11—H11A	120.6
C3—C4—H4A	119.0	C13—C12—C11	121.0 (2)
C5—C4—H4A	119.0	C13—C12—H12A	119.5
C4—C5—C6	118.6 (2)	C11—C12—H12A	119.5
C4—C5—C7	118.7 (2)	C12—C13—C8	122.1 (2)
C6—C5—C7	122.7 (2)	C12—C13—H13A	119.0
C1—C6—N1	120.3 (2)	C8—C13—H13A	119.0
			
C6—C1—C2—C3	0.0 (4)	C6—N1—C8—C13	22.6 (4)
C1—C2—C3—C4	−0.6 (4)	C6—N1—C8—C9	−160.5 (2)
C2—C3—C4—C5	1.1 (4)	N1—C8—C9—C10	−176.0 (2)
C3—C4—C5—C6	−1.1 (4)	C13—C8—C9—C10	1.1 (3)
C3—C4—C5—C7	−179.1 (2)	N1—C8—C9—N2	4.1 (4)
C2—C1—C6—N1	175.9 (2)	C13—C8—C9—N2	−178.8 (2)
C2—C1—C6—C5	0.0 (4)	O3—N2—C9—C10	165.3 (2)
C8—N1—C6—C1	34.3 (4)	O4—N2—C9—C10	−14.0 (4)
C8—N1—C6—C5	−149.9 (2)	O3—N2—C9—C8	−14.8 (4)
C4—C5—C6—C1	0.5 (3)	O4—N2—C9—C8	165.9 (3)
C7—C5—C6—C1	178.5 (2)	C8—C9—C10—C11	−0.4 (4)
C4—C5—C6—N1	−175.3 (2)	N2—C9—C10—C11	179.5 (2)
C7—C5—C6—N1	2.6 (4)	C9—C10—C11—C12	−1.2 (4)
C4—C5—C7—O2	172.6 (2)	C10—C11—C12—C13	2.1 (4)
C6—C5—C7—O2	−5.4 (4)	C11—C12—C13—C8	−1.4 (4)
C4—C5—C7—O1	−7.2 (3)	N1—C8—C13—C12	176.9 (2)
C6—C5—C7—O1	174.8 (2)	C9—C8—C13—C12	−0.2 (3)

### Hydrogen-bond geometry (Å, °)

**Table:**

*D*—H···*A*	*D*—H	H···*A*	*D*···*A*	*D*—H···*A*
N1—H1A···O3	0.86	2.02	2.636 (3)	128.
O1—H1C···O2^i^	0.82	1.82	2.636 (2)	176.

Symmetry codes: (i) −*x*+1, −*y*+1, −*z*+1.
